# Mutations in variable domains of the HIV-1 envelope gene can have a significant impact on maraviroc and vicriviroc resistance

**DOI:** 10.1186/1742-6405-10-15

**Published:** 2013-06-07

**Authors:** Odalis Asin-Milan, Annie Chamberland, Yi Wei, Alpha Haidara, Mohamed Sylla, Cécile L Tremblay

**Affiliations:** 1 Centre de recherche, Centre hospitalier de l’Université de Montréal (CRCHUM), Montréal, QC, Canada; 2 Department of Microbiology and Immunology, Faculty of Medicine, Université de Montréal, Montréal, QC, Canada; 3 Laboratoire de santé publique du Québec/Institut national de santé publique du Québec (INSPQ), Québec, Canada; 4 CRCHUM – Hôtel-Dieu, Pavillon Jeanne-Mance, bureau 7-355, 3840 rue St-Urbain, Montréal, QC H2W 1T8, Canada

**Keywords:** Maraviroc, Vicriviroc, Resistance, CCR5 Inhibitors

## Abstract

**Background:**

Resistance to CCR5 inhibitors, such as maraviroc and vicriviroc is characterized by reduction of maximal percent inhibition which indicates the use of an inhibitor-bound conformation of CCR5 for human immunodeficiency virus-1(HIV-1) entry. It is accompanied by substitutions in gp120 and gp41. Variable domain 3 (V3) plays the most important role, but substitutions outside V3 could also be involved in phenotype resistance. In this work, we investigated how mutations in variable regions of the viral envelope protein gp120 can contribute to CCR5 inhibitor resistance.

**Methods:**

Resistant isolates were selected by passaging CC1/85 and BaL viruses with sub-inhibitory MVC and VCV concentrations. Mutations in gp160 were identified and mutants containing V2 (V169M), V3 (L317W) and V4 (I408T) were constructed.

**Results:**

MVC and VCV susceptibility and viral tropism were assessed by single cycle assay. Mutant I408T showed 4-fold change (FC) increase in the half maximal inhibitory concentration (IC_50_) to MVC, followed by L317W (1.52-FC), V169M (1.23-FC), V169M/I408T (4-FC) L317W/I408T (3-FC), V169M/L317W (1.30-FC), and V169M/L317W/I408T (3.31-FC). MPI reduction was observed for mutants I408T (85%), L317W (95%), V169M/I408T (84%), L317W/I408T (85%) and V169M/L317W/I408T (83%). For VCV, I408T increased the IC_50_ by 2-FC and few mutants showed MPI reduction less than 95%: I408T (94%), L317W/I408T (94%) and V169M/L317W/I408T (94%). All mutants remained R5-tropic and presented decreased infectivity.

**Conclusions:**

These results suggest that mutations in the V4 loop of HIV-1 may contribute to MVC and VCV resistance alone or combined with mutations in V2 and V3 loops.

## Background

HIV-1 entry into target cells is initiated by interactions between the viral envelope (Env) protein gp120 and the host cell receptor CD4. It triggers conformational changes in gp120, forming the co-receptor binding site [1-3]. gp120 interaction with C-C chemokine receptor 5 (CCR5) or C-X-C chemokine receptor 4 (CXCR4) induces other conformational changes in gp120, which evoke structural re-arrangement of gp41 and enables the viral and cellular membrane fusion, permitting viral entry [4]. CCR5 inhibitors, including maraviroc (MVC), vicriviroc (VCV), aplaviroc, TAK-779 and TAK-220, antagonize this process and have strong anti-viral activity against HIV-1 *in vitro*[5,6]. Although they bind the hydrophobic pocket within transmembrane domains of CCR5 with high affinity, they occupy different sub-cavities by interacting with different amino acids [6]. MVC is the first CCR5 inhibitor approved for the treatment of R5-tropic HIV-1 infection in both naïve and treatment-experienced adult patients. VCV development was stopped because of suboptimal efficacy [5]. Since MCV and VCV are allosteric inhibitors of virus entry, resistance to these drugs is evidenced by reduction in the plateau of virus inhibition curves rather than by increases in 50 percent inhibitory concentration (IC_50_) [7,8]. The magnitude of this decrease can be expressed as maximum plateau inhibition (MPI) [9]. Plateau height depends on the relative affinity of HIV-1 for inhibitor-bound versus free CCR5, the greater the affinity for inhibitor-bound CCR5, the lower the height of the plateau [7]. MOTIVATE clinical trials of MVC revealed that the MPI of most MVC-resistant viruses in subjects failing therapy ranged from 80 to 95% [9]. In VICTOR-E1 clinical trials of VCV, phenotypic resistance was manifested by reductions in relative MPI. The cut-off value was 0.94 [8]. Changes in susceptibility to CCR5 inhibitors are usually accompanied by substitutions in gp120, with V3 domain appearing to play a critical role. However, substitutions outside this region also contribute to the resistance phenotype [10]. The aim of this study is to investigate how mutations in other variable loops of the HIV-1 Env can contribute to MVC and VCV resistance.

## Results

### Selection of resistance mutations by *in vitro* passage

After 4 passages of CC1/85 virus in the presence of sub-inhibitory MVC concentrations, some mutations, such as V169M and N192K in V2, L317W in V3, I408A in V4, D462N, N463T, S464T and N465aD in V5, and L820I, I829V and Y837C in gp41,were associated with increased p24 levels (Table 1). After 16 passages, 2 new mutations materialized: I408T in V4 and P849Q in gp41 (Table 1). It is noteworthy that the mutation I408A in V4 appeared after 4 passages and disappeared at passage 16, when a new mutation, I408T, surfaced at the same position.. For VCV, 2 mutations emerged after 4 passages: V169M in V2 and L317W in V3. After 16 passages, the mutation I840Y arose in gp41. No amino acid change was observed with either drug in the Env region of laboratory-adapted BaL virus after 16 passages (Additional file 1).

**Table 1:** Summary of sequence changes in env gene associated with decreased susceptibility to MVC and VCV in passages experiments

**Virus, drug**	**Passage no.**	**gp 120 V2 loop amino acid sequence**
CC1/85	start	158 198
		SFNITTSIRNKVQKQYALFYKLDVVPIDNDSNNTNYRLISC
CC1/85 control	4	------------------------------------------------------------------------
CC1/85, maraviroc	4	--------------------**M**--------------------------------------------**K**-------
CC1/85, vicriviroc	4	--------------------**M**------------------------------------------------------
CC1/85 control	16	-----------------------------------------------------------------------------
CC1/85, maraviroc	16	--------------------**M**--------------------------------------------**K**-------
CC1/85, vicriviroc	16	--------------------**M**------------------------------------------------------
		**gp 120 V3 loop amino acid sequence**
CC1/85	start	296 331
		CTRPNNYTRKSIHIGPGRALYATGDIIGDIRQAHC
CC1/85 control	4	-----------------------------------------------------------------
CC1/85, maraviroc	4	-----------------------------------**W**--------------------------
CC1/85, vicriviroc	4	-----------------------------------**W**--------------------------
CC1/85 control	16	-----------------------------------------------------------------
CC1/85, maraviroc	16	-----------------------------------**W**--------------------------
CC1/85, vicriviroc	16	-----------------------------------**W**---------------------------
		**gp 120 V4 loop amino acid sequence**
CC1/85	start	385 418
		CNSTKLFNSTWTWNNSTWNNTKRANDIEEIITLPC
CC1/85 control	4	--------------------------------------------------------------------
CC1/85, maraviroc	4	-----------------------------------------------------**A-**------------
CC1/85, vicriviroc	4	---------------------------------------------------------------------
CC1/85 control	16	-----------------------------------------------------**A**-------------
CC1/85, maraviroc	16	-----------------------------------------------------**T**--------------
CC1/85, vicriviroc	16	--------------------------------------------------------------------
		**gp 120 V5 loop amino acid sequence**
CC1/85	start	461 469
		KDNSTNEIFR
CC1/85 control	4	-----------------
CC1/85, maraviroc	4	**TN**--**T**----**N**---
CC1/85, vicriviroc	4	-------------------
CC1/85 control	16	---------------
CC1/85, maraviroc	16	**TNTT**-**D**----
CC1/85, vicriviroc	16	-------------
		**gp 41 amino acid sequence**
CC1/85	start	642 681
		IYNLLEESQNQQEKNEQELLELDKWANLWNWFDISNWLWY
CC1/85 control	4	---------------------------------------------------------------------------------
CC1/85, maraviroc	4	--------------------------------------------------------------------------------
CC1/85, vicriviroc	4	--------------------------------------------------------------------------------
CC1/85 control	16	---------------------------------------**A**--------------------------------------
CC1/85, maraviroc	16	---------------------------------------**A**--------------------------------------
CC1/85, vicriviroc	16	--------------------------------------------------------------------------------
		**gp 41 amino acid sequence**
CC1/85	start	812 850
		IKIFINATALAVAEGTDRILEVLQRAYRAILHIPRRIRP
CC1/85 control	4	-----------------**I**-----------------------------------------------------
CC1/85, maraviroc	4	-----------------**I**-----------------**V**-----------**C**--------------------
CC1/85, vicriviroc	4	------------------------------------------------------------------------
CC1/85 control	16	-----------------**I**-----------------**V**----**A**-----**C**---------------------
CC1/85, maraviroc	16	-----------------**I**-----------------**V**-----------**C**-------------------**Q**
CC1/85, vicriviroc	16	------------------------------------------**A**----------**Y**---------------

Residues are numbered according to the HXB2 gp120 sequence.

### Susceptibility of mutant clones to MVC and VCV

**Figure 1:**
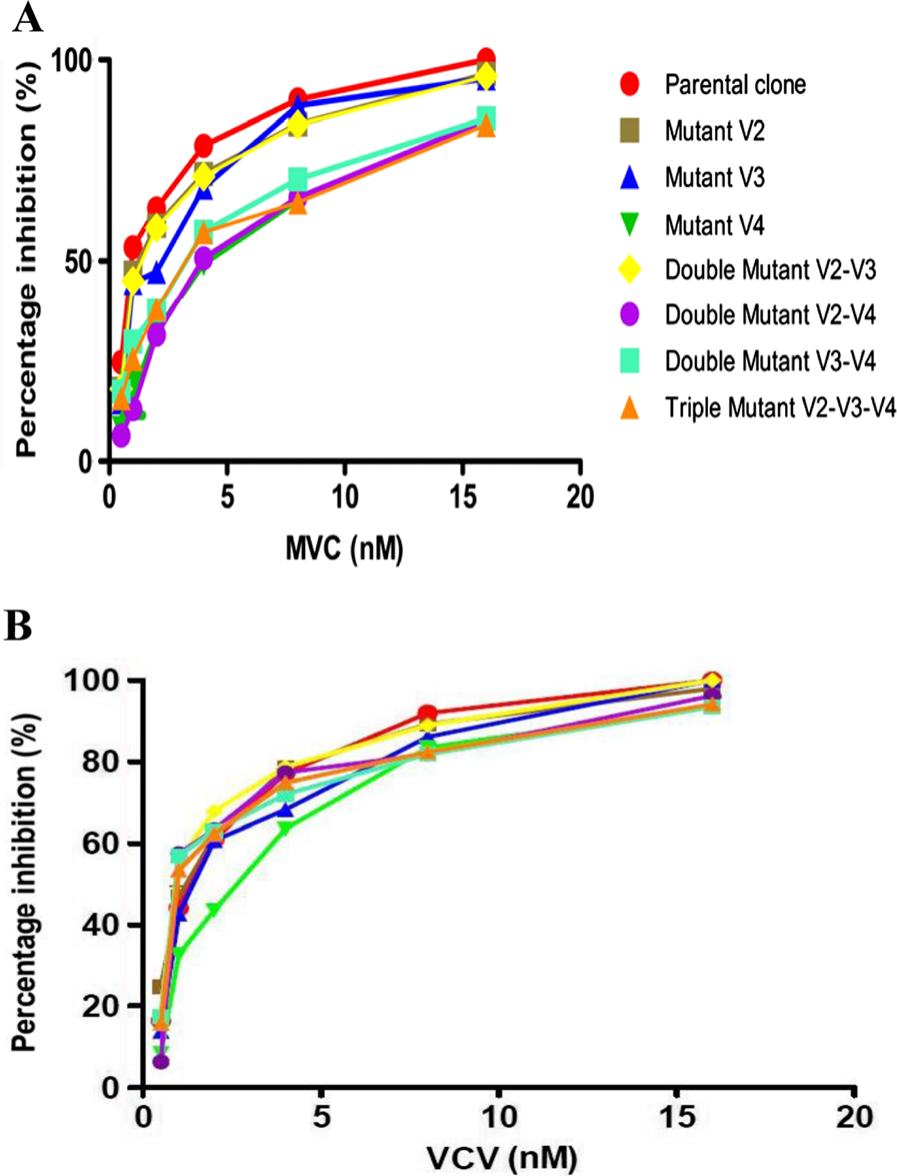
**Sensitivity of parental and mutant clones to MVC (A) and VCV (B).** HIV-1 Env pseudoviruses bearing the parental clone or Env-selected mutations V2 (V169M), V3 (L317W) and V4 (I408T) in single, double and triple combinations served to infect U87-CD4-CCR5 cells in the presence of increasing MVC and VCV concentrations. Infectivity was assessed by measuring luciferase activity 3 days after infection. The GraphPad Prism program generated inhibition curves. In each graph, the percentages of inhibition of parental clones are shown in red, and all results are the means of 3 experiments, each performed in triplicate.

Significant (85%) reduction in MVC's MPI was apparent for I408T, 84% for the double mutant V169M/I408T, 85% for L317W/I408T, and 83% for the triple mutant V169M/L317W/I408T. L317W and V169M/L317W mutants presented intermediate resistance of 95% and 94%, respectively. No significant MPI diminution occurred for the single mutant V169M (96%). A similar pattern was observed when measuring fold-change (FC) increase in the IC_50_, with I408T showing 4-FC, L317W 1.52-FC, and V169M, 1.23-FC increase. The double mutants V169M/L317W exhibited 1.30-FC, V169M/I408T 4-FC and L317W/I408T 3-FC, and 3.31-FC was evident for the triple mutant V169M/L317W/I408T (Table 2 and Figure 1A). The single mutants V169M and L317W and the double mutants V169M/L317W and V169M/I408T retained their susceptibility to VCV, reaching 100% inhibition in some cases. For VCV, I408T increased the IC_50_ by 2-FC, and the mutants I408T, L317W/I408T and V169M/L317W/I408T showed MPI of 94%, 93% and 94%, respectively (Table 3 and Figure 1B).

**Table 2:** Susceptibility and phenotypic properties of V2, V3 and V4 single, double and triple mutants against maraviroc

**Loop**	**Mutant**	**Tropism**	**IC**_**50**_	**IC**_**50**_	**MPI**
				**FC increase**	
**Parental clone**	**Wild type**	**R5**	**1.47 nM**	**-**	**-**
**V2**	**V169M**	**R5**	**1.82 nM**	**1.23**	**96%**
**V3**	**L317W**	**R5**	**2.24 nM**	**1.52**	**95%**
**V4**	**I408T**	**R5**	**5.46 nM**	**4.00**	**85%**
**V2-V3**	**V169M**	**R5**	**1.92 nM**	**1.30**	**94.3%**
	**L317W**				
**V2-V4**	**V169M**	**R5**	**5.76 nM**	**4.00**	**84%**
	**I408T**				
**V3-V4**	**L317W**	**R5**	**4.29 nM**	**3.00**	**85%**
	**I408T**				
**V2-V3-V4**	**V169M**	**R5**	**4.88 nM**	**3.31**	**83%**
	**L317W**				
	**I408T**				

IC_50_, 50% effective concentration or concentration needed to inhibit 50% of HIV. IC_50_ FC was calculated as the ratio IC_50_ for resistant virus/IC_50_ for wild type virus. MPI (maximum percent inhibition) was calculated as described in Methods.

**Table 3:** Summary of susceptibility and phenotypic properties of V2, V3 and V4 single, double and triple mutants against vicriviroc

**Loop**	**Mutant**	**Tropism**	**IC**_**50**_	**IC**_**50**_	**MPI**
				**FC increase**	
**Parental clone**	**Wild type**	**R5**	**1.56 nM**	**-**	**-**
**V2**	**V169M**	**R5**	**1.61 nM**	**1.03**	**100%**
**V3**	**L317W**	**R5**	**1.90 nM**	**1.21**	**100%**
**V4**	**I408T**	**R5**	**3.16 nM**	**2.02**	**94%**
**V2-V3**	**V169M**	**R5**	**1.56 nM**	**1.57**	**100%**
	**L317W**				
**V2-V4**	**V169M**	**R5**	**1.62 nM**	**1.03**	**96%**
	**I408T**				
**V3-V4**	**L317W**	**R5**	**1.68 nM**	**1.07**	**93%**
	**I408T**				
**V2-V3-V4**	**V169M**	**R5**	**1.59 nM**	**1.01**	**94%**
	**L317W**				
	**I408T**				

IC_50_, 50% effective concentration or concentration needed to inhibit 50% of HIV. IC_50_ FC was calculated as the ratio IC_50_ for resistant virus/IC_50_ for wild type virus. MPI (maximum percent inhibition) was calculated as described in Methods.

### Mutations affect virus infectivity differentially

**Figure 2:**
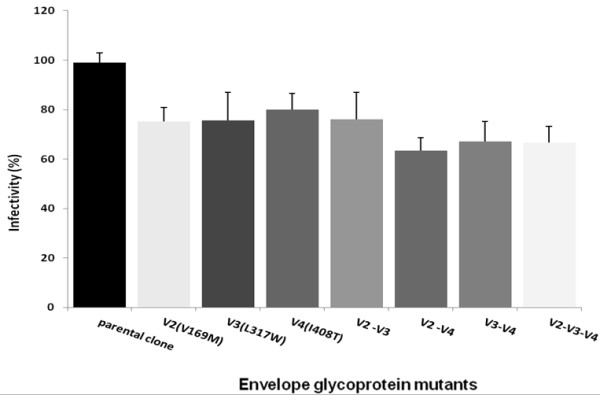
**Effect of Env glycoprotein mutations on infectivity of the HIV-1 strain CC1/85.** U87-CD4-CCR5 cells were infected with HIV-1 Env pseudoviruses bearing parental clone (wild type) or Env single mutants V169M, L317W, I408T, double mutants V169M/L317W, V169M/I408T, L317W/I408T and the triple mutant V169M/L317W/I408T. Means from 3 independent experiments performed in triplicate are reported. Pseudovirus infectivity was calculated as RLU of the mutant/RLU of the wild type × 100%.

The CC1/85 parental clone and mutants exhibited different levels of infectivity. All mutants manifested decreased infectivity that ranged from 20 to 38%. Infectivity percentage was 75% for V169M, 76% for L317W, 80% for I408T, 76% for the double mutants V169M/L317W, 63% for V169M/I408T, 67% for L317W/I408T, and 67% for the triple mutant V169M/L317W/I408T (Figure 2).

### Tropism and V3 net charge

All CC1/85-derived mutants remained R5-tropic. None of them was able to replicate in U87CD4CXCR4+ cells, but did so well in U87CD4CCR5+ cells (Tables 2 and 3). R5-tropism was also confirmed by 2 different genotypic predictors, PSSM_X4R5_[11,12] and geno2pheno co-receptor [13]. No change in V3 net charge was observed.

## Discussion

Our findings indicate that viruses resistant to MVC can retain the use of CCR5 coreceptor as reported previously [14-16]. CCR5 inhibitors are associated with mutations in the Env V3 region of R5 isolates [14,17]. Our passage experiments revealed only 1 polymorphism in the V3 loop crown, L317W, which was associated with reduced infectivity, but not with resistance to CCR5 inhibitors or changes in V3 net charge (Tables 1 and 2). The selection of HIV-1 resistance to CCR5 inhibitors is relatively difficult [14,18], the V3 loop being the least variable of the HIV-1 Env variable regions [19]. Marozsan et al. found no amino acid changes in the V3 loop of CC1/85 resistant to VCV generated *in vitro*[15]. Wesby et al. reported a MVC-resistant CC1/85 virus generated in passage experiments with only 2 changes in amino acid positions 316 and 323 in the V3 loop [14]. Anastassopoulou et al. described D1/86.16, a VCV escape mutant that has no mutations in V3 [20].

Interestingly, when V3 substitution L317W is associated with V4 substitution I408T or triple substitution V169M/L317W/I408T, it confers further reduction of infectivity to 70% of the parental clone (Figure 2). This decreased infectivity could be due to lower fusion activity or binding affinity to the CCR5 co-receptor molecule of the mutant L317W.

The same polymorphism was found by Yu et al. in V3 region of the gp120 isolates CRF07_BC and CRF01_AE, the predominant HIV-1 subtypes in China in patients naïve to CCR5 inhibitors. In their study, R5 isolates harboring 2 dominant polymorphisms, R315Q and F317W in V3, were less susceptible than B isolates to MVC and TAK779. They postulated that baseline resistance to entry inhibitors may be associated with naturally-occurring polymorphisms [21]. Yuan et al. [22] studied a combination of polymorphisms in the gp120 V3 loop of HIV-1JR-FL virus that can confer non-competitive resistance to MVC with a V3 loop library containing a set of random combinations of 0-10 polymorphic mutations *in vitro*. After 17 passages, they found pseudoviruses containing the polymorphism F317W (F312W from the V3 library) with typical non-competitive resistance to MVC. The polymorphism F317W was also found by Muñoz-Nieto et al. in 2008 in the env region from primary isolates during follow-up of dual natural infection with subtypes B and G [23].

In our study, I408T in V4 was the mutation that conferred the highest level of resistance. This mutation occurred only in viruses resistant to MVC in passage experiments. It was a change from medium size and hydrophobic I to medium size and polar T. The mutation conferred resistance to MVC and was linked with cross-resistance to VCV, as indicated by MPI values. Although cross-resistance was observed for VCV, the virus was more resistant to MVC. Cross-resistance is commonly seen among CCR5 inhibitors [5]. Even if VCV had a favorable resistance profile, its virological activity was sub-optimal in phase II and III studies, possibly because of its pharmacokinetic profile and the dose chosen [5,24]. Tilton et al. [25] analyzed viruses from 11 treatment-experienced patients in whom virologic failure occurred on regimens containing MVC and noted that resistance was dependent upon mutations within the V3 loop which was only modulated by additional mutations in the V4 loop. The V4 mutations consisted of D407G and loss of a glycosylation site at residue 386.

Platt et al. [26] described HIV-1 CCR5 (∆Nt)-adapted mutants of the JR-CSF strain that had mutations in regions V3, V2 and C3 with 4 mutations in the V4 loop: N403S, N403K, T405A, and T405N. They concluded that loss of N-glycan at position 403 helps to convert the HIV-1 env into a hairpin-trigger form that no longer requires strong interactions with both the CCR5 amino terminus and ECL2 but efficiently targets either site alone [27-32]. Based on these models, we could postulate that the presence of T in position 408 of the V4 loop could alter the quaternary structure of the gp120-gp41 trimeric complex, eliciting conformational transition from a high-energy to a low-energy state (fusogenic conformation), enhancing membrane fusion, and promoting the next step in the virus entry pathway. Since positions 403 and 408 of the V4 loop are close proximity, we imagine that the resistance conferred by I408T mutation in our study could alter the quaternary structure of the HIV-1 Env, thus sterically masking the glycosylation site in position 403.

Mutations in V2, C3 and gp41 have also been observed in CC1/85-derived, MVC-resistant viruses [14]. The mutation V169M, identified in our study, was also found by Marozsan et al. in VCV escape mutant, which was resistant to VCV, but they did not investigate the contribution of each individual mutation to the resistant phenotype [15]. Recently, Thielen et al. [33] demonstrated that mutations at position 169 of the V2 loop can serve in HIV-1 co-receptor prediction. In their study, the mutation V169T showed strong association with CXCR4 usage while V169K was coupled with CCR5 usage. In another study, the mutation V169K in V2 was predictive of the R5 phenotype [34]. Our results disclosed that mutant V169M exploits R5 exclusively as a co-receptor. V169M mutation was coupled with reduced infectivity but not with a resistant phenotype. Unexpectedly, our triple mutant was less infective than single and double mutants as the emergence of mutations throughout gp120 and gp41 indicated general envelope gene (env) re-arrangement to compensate for decreased replicative capacity [35,36].

## Conclusions

Several mutations outside the V3 loop were shown to contribute to CCR5 inhibitor resistance. Our results showed that I408T, L317W/I408T and V169M/L317W/I408T mutants had the highest impact on MVC susceptibility, mostly due to I408T in V4. This mutation could lower the activation energy needed to enable gp41 to undergo the next conformational changes and acquire a more stable low-energy state. All mutants retained the CCR5 co-receptor, supporting the concept that resistant viruses maintained the ability to use inhibitor-bound CCR5, depending on co-receptor density on the cellular surface and the degree of CCR5 co-receptor occupancy by drugs.

## Methods

### Compounds, cells and viruses

MVC and VCV were obtained from ViroChem Inc. Canada, now Vertex Canada Inc., reconstituted in DMSO 100% (Sigma, St. Louis, MO, USA) and diluted in RPMI 1640 culture medium (Sigma). Peripheral blood mononuclear cells (PBMCs) from 3 HIV-negative donors were isolated by Ficoll-Paque gradient separation (Sigma), stimulated for 3 days with phytohemagglutinin (Sigma) at 1 mg/ml and re-suspended at 2 × 10^6^ cells/ml in RPMI 1640 supplemented with 20% heat-inactivated fetal calf serum (FCS, Invitrogen, Carlsbad, CA, USA), 50 U/ml penicillin (Invitrogen), 50 μg/ml streptomycin (Invitrogen)., 2 mM L-glutamine, 10 mM hydroxyethylpiperazine N-2 ethanesulfonic acid (HEPES) and 1 μg/ml interleukin-2 (Hoffmann-La Roche, Nutley, NJ, USA) in 6-well plates (Becton Dickinson, Lincoln Park, N.J USA.). 293T cells were cultured in Dulbecco’s modified Eagle medium (DMEM) and supplemented with 10% FCS, 100 U/ml penicillin and 0.1 mg of streptomycin/ml U87CD4CXCR4 and U87CD4CCR5 cells were cultured in DMEM and supplemented with 10% FCS, 100 U/ml of penicillin, 0.1 mg of streptomycin/ml, 1 μg/mL of puromycin (Sigma) and 300 μg of G418 (Invitrogen). All cells were maintained at 37°C and 5% CO_2_. Cells and the viruses BaL and CC1/85 (R5 tropic), III B (X4-tropic) and 85.6 (dual-tropic) were obtained from the National Institutes of Health.

### Generation of HIV-1 mutants resistant to MVC and VCV by *in vitro* passage in PBMCs

2 × 10^6^ PBMCs/ml were infected with 3,000 tissue culture infective doses 50% of BaL and CC1/85 viruses, in a final volume of 2 ml containing sub-inhibitory concentrations of 0.04 nM MVC and 0.02 nM VCV. Every 7 days, the culture supernatant was passaged onto fresh cells with MVC- and VVC-containing medium, and virus growth was monitored by enzyme-linked immunosorbent assay (ELISA) to measure supernatant p24 levels (Perkin-Elmer, Norwalk, CT, USA). MVC and VCV were added every 4 days at concentrations depending on p24 levels. MVC and VCV control passages were set up in parallel. Full length sequences of gp120 and gp41 were obtained for different time points, and mutations from resistant viruses were identified. Resistance was defined as MPI <95% or ≥3-FC in IC_50_. Viral tropism was predicted according to 11/25 and net charge rules [37] as well as PSSM_X4R5_[11,12] and geno2pheno co-receptor algorithms [13].

### Amplification and sequence analysis of the HIV env gene from selected time-points

HIV-1 RNA was extracted from culture supernatants with QIAmp Viral RNA mini-kit (Qiagen, Mississauga, ON, Canada). Full-length gp160 was amplified with reverse transcription -polymerase chain reaction (RT-PCR) primers (5’ NewFHindIII GGCCAAGCTTATGAGAGTGACGGAGATCAG and 5’ YW15XhoI GGCCCTCGAGTTATCCAGTCCCCCCTTTTC), followed by nested polymerase chain reaction (PCR) with primers (5’ NewFHindIII GGCCAAGCTTATGAGAGTGACGGAGATCAG and 5’ YW16XhoI GGCCCTCGAGTTATTTTGACCACTTGCCAC). PCR products were separated on 1% agarose gel and purified with QIAprep Spin Miniprep Kit (Qiagen). Sequencing was undertaken at the Génome-Québec sequencing facilities in a 3730 × l DNA analyser from Applied Biosystems (McGill University and the Genome Quebec Innovation Centre, Montreal, QC, Canada) with Sequencer 4.7 (Gene Code Software Corporation, Ann Arbor, MI, USA) and aligned by ClustalW version 1.83 [10]. Nucleotide sequences of CC1/85 have been deposited in GenBank under accession numbers JQ924495 (start), JQ924496 (controlP4), JQ924497 (MVCP4), JQ924498 (VCVP4), JQ924499 (controlP16), JQ92450 (MVCP16), and JQ924501 (VCVP16).

### Site-directed mutagenesis

Primers were designed with the Stratagene’s web-based QuikChange® Primer Design Program [38]. Mutagenesis procedures were carried out according to an overlapping-extension PCR-based procedure [39,40].

### Cloning and pseudovirus construction

The expression plasmid pcDNA3.1day/V5HisTOPO (Invitrogen) and Env PCR products of CC1/85, BaL, III B, 85.6 viruses and selected mutants (V169M in V2, L317W in V3 and I408T in V4) in single, double and triple combinations were digested with HindIII and Xho1 restriction enzymes (Invitrogen), purified and ligated with T4 DNA Ligase (Invitrogen). The ligation product was transformed into *E. coli* TOP10 competent cells. Pseudoviruses were produced by co-transfection of Env expression plasmid and backbone pNL4-3. Luc.E-R-, as described previously [41,42]. Pseudovirus stocks were normalized with p24 ELISA prior to testing infectivity. Assays were performed with 25 ng of p24 per well.

### Luciferase assays to determine infectivity and tropism

A luminescence assay using U87CD4CXCR5 and U87CD4CXCR4 cells was used to measure infectivity and tropism. On the day prior to infection, 1×10^6^ cells per well were seeded in 96-well plates. On the day of infection, MVC and AMD 3100 were added to wells designated for treatment with an inhibitor and incubated for 1 h at 37°C prior to infection. 50 μl of normalized pseudovirus stocks were added in each well. The plates were incubated for 2 days at 37°C with 5% CO_2_. The medium was removed, and 100 μl of lysis buffer (Promega Inc., Madison, WI, USA) was added to each well for 30 min. Then, 100 μl of luciferase assay reagent (Promega) was added immediately prior to reading the plates in a luminometer (Tecan, Morrisville, NC, USA). Luciferase activity was recorded as relative light units (RLU). Viral entry was determined as percent reduction of viral infectivity compared to the controls. All experiments were performed in duplicate. IC_50_-FC was calculated as the ratio of IC_50_ for resistant virus/IC_50_ for wild type virus. MPI was calculated as [1- (RLU in the presence of drug/RLU in the absence of drug)] × 100, and infectivity as RLU of the mutant/RLU of the wild type × 100%. Inhibition curves were generated by GraphPad Prism software (San Diego, CA, USA).

## Abbreviations

(HIV-1): Human immunodeficiency virus-1 entry; (V3): Variable domain 3; (IC50): Half maximal inhibitory concentration; (Env): Envelope protein; (CCR5): C-C chemokine receptor 5; (CXCR4): C-X-C chemokine receptor 4; (MVC): Maraviroc; (VCV): Vicriviroc; (MPI): As maximum percent inhibition; (FC): Measuring fold-change; (env): Envelope gene.

## Competing interests

CT is the Pfizer/University of Montreal Chair in HIV Translational Research and a scholar from Fonds de la recherche en santé du Québec. The other authors declare no conflicts of interest.

## Authors’ contributions

OA-M participated in study conception and design, data collection, analysis and interpretation as well as manuscript drafting; AC supervised the study, analyzed and interpreted the data, and reviewed the manuscript; YW participated in study conception and design; AH participated in data collection; MS participated in data collection, analysis and interpretations; CT participated in study conception and design, data analysis and interpretation, study supervision, and manuscript review. All authors read and approved the final manuscript.

## Supplementary Material

Additional file 1 The Env region of laboratory-adapted BaL virus after 16 passages in the presence of sub-inhibitory VCV concentrations. Click here for file
