# Supplementary material for: Mutations in variable domains of the HIV-1 envelope gene can have a significant impact on maraviroc and vicriviroc resistance
Source: AIDS Res Ther. 2013 Jun 7;10:15. doi: 10.1186/1742-6405-10-15 (PMC3700831; doi:10.1186/1742-6405-10-15)
Supplement: Additional file 1 — The Env region of laboratory-adapted BaL virus after 16 passages in the presence of sub-inhibitory VCV concentrations. [file 1742-6405-10-15-S1.docx]

**Additional File 1.** **The Env region of laboratory-adapted BaL virus after 16 passages in the presence of sub-inhibitory VCV concentrations**.

| **Virus, drug** | **Passage No.** | **gp 120 V2 loop amino acid sequence** |
| --- | --- | --- |
| Bal | start | 158 198  SFKIVTNIRGKVKKEYALFHELDIVPIDNKNDNYRLISC |
| BAL control | 4 | ---------------------------------------- |
| BAL, maraviroc | 4 | ---------------------------------------- |
| BAL, vicriviroc | 4 | ---------------------------------------- |
| BAL control | 16 | ---------------------------------------- |
| BAL, maraviroc | 16 | ---------------------------------------- |
| BAL, vicriviroc | 16 | ---------------------------------------- |
|  |  | **gp 120 V3 loop amino acid sequence** |
| BAL | start | 296 331  CTRPNNYTRKSIHIGPGRALYPIGEIIGDIRQAHC |
| BAL control | 4 | ----------------------------------- |
| BAL, maraviroc | 4 | ----------------------------------- |
| BAL, vicriviroc | 4 | ----------------------------------- |
| BAL control | 16 | ----------------------------------- |
| BAL, maraviroc | 16 | ----------------------------------- |
| BAL, vicriviroc | 16 | ----------------------------------- |
|  |  | **gp 120 V4 loop amino acid sequence** |
| BAL | start | 385 410  CSSNITGLLESNNTVENNTITLP |
| BAL control | 4 | ---------------------- |
| BAL, maraviroc | 4 | ---------------------- |
| BAL, vicriviroc | 4 | ---------------------- |
| BAL control | 16 | ---------------------- |
| BAL, maraviroc | 16 | ---------------------- |
| BAL, vicriviroc | 16 | ---------------------- |
|  |  | **gp 120 V5 loop amino acid sequence** |
| BAL | start | 461 469  EDNKTEVFR |
| BAL control | 4 | ---------- |
| BAL, maraviroc | 4 | ---------- |
| BAL, vicriviroc | 4 | ---------- |
| BAL control | 16 | ---------- |
| BAL, maraviroc | 16 | ---------- |
| BAL, vicriviroc | 16 | ------------- |
|  |  | **gp 41 amino acid sequence** |
| BAL | start | 642 681  IYSLIEESQNQQEKNEQELLELDKWASLWNWFDITKWLWY |
| BAL control | 4 | ---------------------------------------- |
| BAL, maraviroc | 4 | ---------------------------------------- |
| BAL, vicriviroc | 4 | ---------------------------------------- |
| BAL control | 16 | ---------------------------------------- |
| BAL, maraviroc | 16 | ---------------------------------------- |
| BAL, vicriviroc | 16 | ---------------------------------------- |
|  |  | **gp 41 amino acid sequence** |
| BAL | start | 812 845  SAVSLLNTIAIAVAEGTDRVIEVVQRAVRAILHX |
| BAL control | 4 | ----------------------------------- |
| BAL, maraviroc | 4 | ----------------------------------- |
| BAL, vicriviroc | 4 | ----------------------------------- |
| BAL control | 16 | ----------------------------------- |
| BAL, maraviroc | 16 | ----------------------------------- |
| BAL, vicriviroc | 16 | ----------------------------------- |

**Legend:** Residues are numbered according to the HXB2 gp120 sequence.
